# Freezing tolerance and tolerance to de-acclimation of European accessions of winter and facultative barley

**DOI:** 10.1038/s41598-023-47318-y

**Published:** 2023-11-15

**Authors:** Magdalena Wójcik-Jagła, Marcin Rapacz

**Affiliations:** Department of Plant Breeding, Physiology and Seed Science, University of Agriculture, Krakow, Poland

**Keywords:** Photosynthesis, Plant physiology, Plant stress responses

## Abstract

Due to global warming, winter hardiness may seem to become less important for plant survival and yield. However, this is a superficial assumption, as probably only the most important factors locally affecting plant overwintering will change. For example, the frequency, degree, and length of extreme winter warming events may increase, leading to de-acclimation of plants. This study aimed to investigate existing variability in de-acclimation tolerance in Polish winter barley breeding materials and European winter and facultative barley cultivars, and to identify accessions with the highest and the lowest tolerance to de-acclimation by means of visual estimation of regrowth after freezing, measurements of electrolyte leakage and chlorophyll fluorescence, and LT50 assessment. The results of this study showed that freezing tolerance and tolerance to de-acclimation are independent traits, and even highly freezing tolerant plants can be susceptible to de-acclimation. Our results highlight the role of photosynthetic apparatus in de-acclimation, proving that chlorophyll fluorescence parameters, especially ET_0_/CS, can be useful indicators of tolerance to de-acclimation. This study also confirmed that although the mechanisms of response to de-acclimation seem to be common for susceptible barley accessions, the mechanisms of tolerance are different, and may be related to the accession’s origin.

## Introduction

Climate change is the major challenge for the world economy, especially for the agricultural sector, where climate conditions strongly and directly influence crop yields. An increase in surface temperature of the Earth indicates a lower risk of crop exposure to extremely low temperatures. However, the risk of winter damage to the plants may remain at the same level or even increase due to complex interactions among different environmental factors. For example, the frequency, degree, and length of extreme winter warming events may increase, leading to de-acclimation of plants^1,2^.

Winter hardiness is an ability of a plant to survive the winter, and it depends on the plant potential to cope with a wide spectrum of environmental stresses, such as freezing, rapidly changing temperatures, low light intensity, desiccation, wind, snow cover, ice encasement, or various winter-related diseases^3^. Freezing tolerance is the most recognized compound of winter hardiness and, in most cases, it has the biggest impact on plant winter survival. Freezing tolerance is indeed a prerequisite for plant survival during winter in moderate continental and maritime climate, but it is not always sufficient^4–7^. Sometimes different traits determine the level of winter hardiness, which may depend on the course of the season. One of these traits is tolerance to de-acclimation (de-hardening), which largely affects plant winter survival, only if an appropriately long warm spell is followed by frost^1^. Mechanisms involved in plant de-acclimation caused by warm spells during winter are not sufficiently recognized^8,9^. Recently, it has been suggested that oxidoreductases may play an important role in the mechanism of response to de-acclimation in barley^9^, but further investigation is needed. It is unclear, what makes some plants de-harden after only a few warmer days, and why other plants need much more time for that. Also, the mechanisms responsible for high survival rate of some plants after experiencing freezing temperatures following a warm spell are still unknown. These particular plants may not de-harden during the warm spell, but they can also re-harden fast enough. Some plants may be resistant to freezing to some degree, making them capable of surviving a short period of freezing temperatures even when non-acclimated.

Moreover, complete understanding of how plants survive mild winters when exposed to mid-winter de-acclimation, requires studies on the plant re-acclimation when the temperatures in late winter or early spring drop down, as shown by^10,11^.

Winter barley is among the least popular cereals grown in Poland, as it shows the lowest winter hardiness of all winter cereals grown in our country^12^. However, during mild winters, winter barley always performs better with regard to yield than spring barley^13,14^. This shows that the yield potential is high, there are just some obstacles to overcome to breed winter barley with improved winter hardiness. Yield losses due to winter damage are high in winter barley, even in mild and warm winters, which have become more frequent in Poland in recent years^14,15^. It can be assumed that the reason for this is low tolerance of barley to mid-winter de-acclimation.

This study aimed to investigate existing variability in de-acclimation tolerance among Polish winter barley breeding materials and European winter and facultative barley cultivars. We assumed that due to no breeding efforts to achieve tolerance to de-acclimation, variability in this trait among Polish advanced winter barley breeding lines and selected European cultivars is much higher than the variability in freezing tolerance. The study also aimed to identify advanced winter barley breeding lines and European barley cultivars with the highest and the lowest tolerance to de-acclimation, on the basis of physiological measurements and observations made during two subsequent experiments.

## Material and methods

### Plant material and growth conditions

In this study, 24 European cultivars of winter and facultative barley and 34 Polish advanced breeding lines of winter barley were screened for their freezing tolerance (Table 1). The seeds of the European cultivars were obtained from the Italian Council for Agricultural Research and Economics (CREA), while the seeds of the Polish advanced breeding lines were provided by the Plant Breeding Company “Danko”. The collection of plant material used in this study has complied with the relevant institutional, national, and international guidelines and legislation. The plant seeds were sown in plastic boxes in four replicates (randomly chosen rows of 12 seeds in 4 different boxes), in two series (first the European cultivars, then the Polish breeding lines with two European cultivars as control). After sowing, the boxes were transferred to controlled conditions (growth chamber, darkness, 25/17 °C, day/night temperature). Light (irradiance of 400 μmol/(m^2^s) (HPS lamps, SON-T + AGRO, Philips, Brussels, Belgium), photoperiod of 12/12h) was turned on after the seedlings started to emerge. Temperature was lowered to 15/12 °C (day/night) eight days after sowing. At 18 to 20 days after sowing, the plants were cold-acclimated (3 weeks, 4/2 °C, day/night temperature, photoperiod of 9/15 h, and irradiance of 250 μmol/(m^2^s).

**Table 1:** Characteristics of plant material used in the study.

No	Name	Origin	Growth habit	Source
1	Aday-2	Turkey	Winter	CREA, IT
2	Aday-4	Turkey	Winter	CREA, IT
3	Aquirone	Italy	Winter	CREA, IT
4	Astartis	Italy	Winter	CREA, IT
5	Atlante	Italy	Winter	CREA, IT
6	Avci 2002	Turkey	Winter	CREA, IT
7	Aydanhanim	Turkey	Winter	CREA, IT
8	Bruker Stamm II	Austria	Facultative	CREA, IT
9	Carola	Austria	Winter	CREA, IT
10	Colonia	Germany	Winter	CREA, IT
11	Cometa	Italy	Winter	CREA, IT
12	DS. 268/14–1	Poland	Winter	Danko, PL
13	DS. 317/14–1	Poland	Winter	Danko, PL
14	DS. 354/14–1	Poland	Winter	Danko, PL
15	DS. 420/14–1	Poland	Winter	Danko, PL
16	DS. 909/16	Poland	Winter	Danko, PL
17	DS. 911/16	Poland	Winter	Danko, PL
18	DS. 912/16	Poland	Winter	Danko, PL
19	DS. 928/16	Poland	Winter	Danko, PL
20	DS. 929/16	Poland	Winter	Danko, PL
21	DS. 930/16	Poland	Winter	Danko, PL
22	DS. 934/16	Poland	Winter	Danko, PL
23	DS. 935/16	Poland	Winter	Danko, PL
24	DS. 936/16	Poland	Winter	Danko, PL
25	DS. 970/16	Poland	Winter	Danko, PL
26	DS. 976/16	Poland	Winter	Danko, PL
27	DS. 977/16	Poland	Winter	Danko, PL
28	DS. 979/16	Poland	Winter	Danko, PL
29	DS. 980/16	Poland	Winter	Danko, PL
30	DS. 983/16	Poland	Winter	Danko, PL
31	DS. 987/16	Poland	Winter	Danko, PL
32	DS. 989/16	Poland	Winter	Danko, PL
33	DS. 1014/16	Poland	Winter	Danko, PL
34	DS. 1016/16	Poland	Winter	Danko, PL
35	DS. 1017/16	Poland	Winter	Danko, PL
36	DS. 1018/16	Poland	Winter	Danko, PL
37	DS. 1020/16	Poland	Winter	Danko, PL
38	DS. 1022/16	Poland	Winter	Danko, PL
39	DS. 1023/16	Poland	Winter	Danko, PL
40	DS. 1026/16	Poland	Winter	Danko, PL
41	DS. 1028/16	Poland	Winter	Danko, PL
42	DS. 1030/16	Poland	Winter	Danko, PL
43	DS. 1033/16	Poland	Winter	Danko, PL
44	DS. 1035/16	Poland	Winter	Danko, PL
45	DS. 1037/16	Poland	Winter	Danko, PL
46	Explora	Italy	Winter	CREA, IT
47	Frost	Sweden	Winter	CREA, IT
48	Gloria	Poland	Winter	Danko, PL
49	Holmes	Poland	Winter	Danko, PL
50	Karakan	Poland	Winter	Danko, PL
51	Mansholt Fletumer	The Netherlands	Winter	CREA, IT
52	Mellori	France	Winter	CREA, IT
53	Pamina	Germany	Facultative	CREA, IT
54	Quadriga	Poland	Winter	Danko, PL
55	Tiffany	Denmark	Winter	CREA, IT
56	Traminer	Austria	Winter	CREA, IT
57	Vincenta	Germany	Winter	CREA, IT
58	Zenek	Poland	Winter	Danko, PL

### Freezing tolerance assessment

After 3 weeks of cold acclimation, the plants were sampled for freezing tolerance assessment. Survival of the plants after freezing was assessed using three methods: regrowth after freezing (FT-R, similar to^16^), measurement of transient parameters of chlorophyll fluorescence (Table 2) after freezing^17^, and electrolyte leakage (EL), as described in detail by^18^. Before testing, the leaves were cut about 1 cm above the soil. Plastic boxes (containing the rest of the plants after cutting) were put in a programmed freezer. Temperature decreased at a rate of 2 °C/h from 0 to –8 °C, –10 °C, and –12 °C, and after 12 h increased at the same rate. The cut leaves were placed into plastic bags, and put together in a programmed freezer with the batch of plastic boxes meant for freezing at –12 °C. Afterward, the plants were placed in an unheated glasshouse, and the leaves were used for chlorophyll fluorescence measurements. A visual estimation of FT-R was done after 10 days and after 21 days. Each accession in FT-R estimation was represented by 15 to 40 replicates (individual plants growing in four different boxes, 10 seeds/box, diverse germination rate). Induction of chlorophyll *a* fluorescence signal in the defrosted leaves was measured after 30 min of the leaf dark adaptation with the Handy PEA fluorimeter (Hansatech, Kings Lynn, UK). The measurements were performed in 10 replicates (10 leaves from different plants). The EL was measured using two about 2 cm-long fragments cut from the middle part of the second leaf of 10 plants from each cultivar/line and each freezing temperature. The leaf fragments were submerged in deionized water (which conductivity, W, was measured prior to the experiment), and gently shaken for 24 h. Then, the first measurement of conductivity of the water mixed with leaf sap was performed (EL1), followed by putting the leaf fragments in liquid nitrogen to destroy cell walls and membranes, and placing them back in the same liquid they were in before. After 24 h of shaking the destroyed leaf fragments, conductivity of the water mixed with leaf sap was measured again (EL2). EL was calculated as follows: EL = (EL1 – W)/(EL2 – W)100%.

**Table 2:** Formulae and glossary of terms used by the OJIP-test in the present study (modified after Strasser et al. 2004).

Data extracted from the recorded fluorescence transient OJIP	
F_t_	Fluorescence at time t after onset of actinic illumination
F_50µs_ or F_20µs_	Minimal reliable recorded fluorescence, at 50 µs with the PEA- or 20 µs with the Handy-PEA-fluorimeter
F_300µs_	fluorescence intensity at 300µs
F_J_≡F_2ms_	Fluorescence intensity at the J-step (2 ms) of OJIP
F_I_≡F_30ms_	Fluorescence intensity at the I-step (30 ms) of OJIP
F_P_	Maximal recorded fluorescence intensity, at the peak P of OJIP
Fluorescence parameters derived from the extracted data	
F_0_ ≅ F_50µs_ or ≅ F_20µs_	Minimal fluorescence (all PSII RCs are assumed to be open)
F_M_ (= F_P_)	Maximal fluorescence, when all PSII RCs are closed (equal to F_P_ when the actinic light intensity is above 500 μmol photons m^−2^ s^−1^ and provided that all RCs are active as Q_A_ reducing)
F_V_≡F_M_ − F_0_	Maximal variable fluorescence
V_t_ ≡ F_υ_/F_V_≡(F_t_—F_0_)/(F_M_ − F_0_)	Relative variable fluorescence at time t
M_0_ ≡ [(ΔF/Δt)_0_]/(F_M_ − F_50µs_) ≡ 4(F_300µs_ − F_50µs_)/(F_M_ − F_50µs_)	Approximated initial slope (in ms^-1^) of the fluorescence transient normalised on the maximal variable fluorescence F_V_
Specific energy fluxes (per Q_A_-reducing PSII reaction center—RC)	
ABS/RC = M_0_ (1/V_J_)(1/ϕ_Po_)	Absorption flux (of antenna Chls) per RC
TR_0_/RC = M_0_ (1/V_J_)	Trapped energy flux (leading to Q_A_ reduction) per RC
ET_0_/RC = M_0_ (1/V_J_)ψ_Eo_	Electron transport flux (further than Q_A_) per RC
DI_0_/RC = (ABS/RC) − (TR_0_/RC)	Dissipated energy flux, per RC
Quantum yields and efficiencies	
ϕ_Po_ ≡ TR_0_/ABS = Fv/FM	Maximum quantum yield for primary photochemistry
ψ_o_ ≡ ET_0_/TR_0_ = (1 − V_J_)	Efficiency/probability for electron transport (ET), i.e. efficiency/probability that an electron moves further than Q_A_^-^
ϕ_Eo_≡ET_0_/ABS = [1 − (F_0_/F_M_)]ψ_Eo_	Quantum yield for electron transport (ET)
ϕ_Ro_≡RE_0_/ABS = [1 − (F_0_/F_M_)]ψ_Eo_ δ_Ro_	Quantum yield for reduction of end electron acceptors at the PSI acceptor side (RE)
Phenomenological fluxes	
ABS/CS = *F*o or ABS/CSM = *F*M TR_0_/CS = ΦPo·(ABS/CS) ET_0_/CS = ΦPo·Ψo·(ABS/CS) DI_0_/CS = (ABS/CS) − (TRo/CS)	*Absorption per excited cross-section* Trapping per excited cross-section Electron transport per excited cross-section Dissipated energy flux per excited cross-section
Performance indexes (products of terms expressing partial potentials at steps of energy bifurcations)	
PI_ABS_ ≡ [γ_RC_/(1 − γ_RC_ )].[ϕ_Po_/(1 − ϕ_Po_)].[ψ_o_/(1- ψ_o_]	Performance index (potential) for energy conservation from exciton to the reduction of intersystem electron acceptors
PI_total_ ≡ (PI_ABS_).(δ_Ro_/1 − δ_Ro_)	Performance index (potential) for energy conservation from exciton to the reduction of PSI end acceptors

Subscript “0” indicates that the parameter refers to the onset of illumination.

### De-acclimation tolerance assessment

Out of the 58 tested lines and cultivars, 20 that proved to be the most freezing tolerant at –10 °C and –12°C (8 from the group of the Polish breeding lines and 12 of the European cultivars, including one Polish), were further tested for their tolerance to de-acclimation. After 3-week acclimation to cold, the plants were subjected to de-acclimation (7 days at 12/5 °C, day/night), and sampled afterwards for freezing tolerance assessment. Plant survival after freezing was assessed in the same manner as described above, at the freezing temperature of –10 °C.

### LT50 assessment

LT50, as the temperature killing 50% of the plants, was assessed for four lines/cultivars selected as tolerant to de-acclimation, and four selected as susceptible. The seeds were sown, and the plants were acclimated to cold and de-acclimated in the same manner as described above. Plant survival was tested for cold-acclimated and de-acclimated plants, using FT-R method. There were six freezing temperatures: –2, –4, –6, –8, –10, and –12 °C. The plants were frozen for two hours at these temperatures. The plant FT-R was assessed on a 0–9 scale^16^, where 0 referred to plants showing no signs of life, and 9 referred to those showing full regrowth. The score of 4.5 was considered a threshold value, corresponding to 50% of plants killed by freezing. For each accession, about 30 plants were assessed per single temperature (three rows in separate boxes, approx. 10 plants each, depending on the germination rate).

### Statistical analysis

Statistical analysis was performed using Statistica 13 software (Dell, Round Rock, TX). The data were analyzed in ANOVA module with an accession as a single variable for cold-acclimated and de-acclimated samples separately. Primary Component Analysis (PCA) was performed in ‘Multifactorial analysis’ module.

## Results

### Freezing tolerance assessment

**Figure 1:**
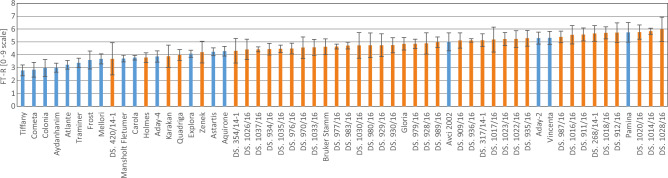
Plant regrowth after freezing (FT-R, scale 0–9) in − 10 °C of 58 barley accessions (Polish breeding lines, marked in orange and European cultivars, marked blue) cold-acclimated for 3 weeks at 4/2 °C (day/night). Means and confidence intervals for *P* = 0.05 (a mean, which is in a range of error bar did not differ statistically at *P* = 0.05). Homogeneity groups (HSD test) are shown in Table S1.

Diversity in the freezing tolerance of the tested accessions was visible after freezing at all temperatures, with the highest mortality rate at − 12 °C (Figure S1). Freezing at − 8 °C poorly diversified the tested lines and cultivars in either series, while the temperature of − 12 °C seemed to be too severe (Figure S1). The temperature of –10 °C provided the optimal freezing conditions for the tests in this group of diverse barley breeding lines and cultivars (Fig. 1).

**Figure 2:**
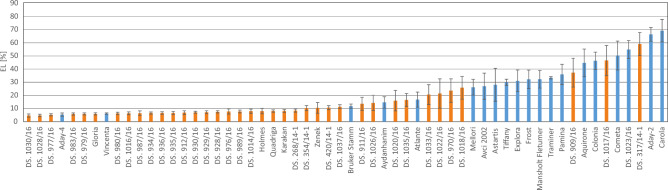
Electrolyte leakage (EL) [%] measured after freezing at − 10 °C on leaves of 58 barley accessions (Polish breeding lines, marked in orange and European cultivars, marked blue) cold-acclimated for 3 weeks at 4/2 °C (day/night). Means and confidence intervals for *P* = 0.05 (a mean, which is in a range of error bar did not differ statistically at *P* = 0.05). Homogeneity groups (HSD test) are shown in Table S1.

**Figure 3:**
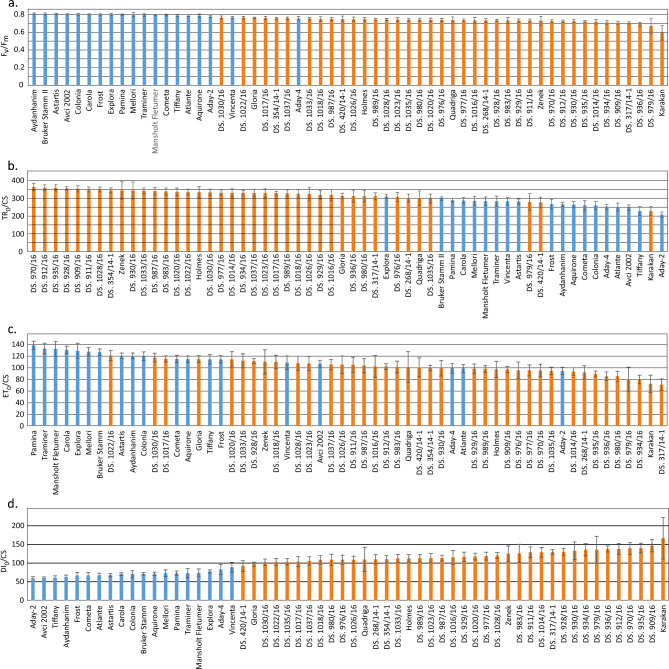
Chlorophyll fluorescence parameters measured after freezing at − 10 °C on leaves of 58 barley accessions (Polish breeding lines, marked in orange and European cultivars, marked blue) cold-acclimated for 3 weeks at 4/2 °C (day/night). Means and confidence intervals for *P* = 0.05 (a mean, which is in a range of error bar did not differ statistically at *P* = 0.05). Homogeneity groups (HSD test) are shown in Table S1.

The results of FT-R, chlorophyll fluorescence, and EL measurements after freezing of the tested lines and cultivars showed considerable diversity in their freezing tolerance, especially at − 10 °C (Figs. 1, 2, 3) and − 12 °C (Figure S1b, S2b). Most of the non-Polish European cultivars displayed lower regrowth rate after freezing than the Polish breeding lines and cultivars (Fig. 1).

In the case of EL, the values measured after freezing at − 8 °C and − 10 °C were rather similar and low (below or around 10%) for ca 75% of the accessions (Figs. 2 and S2a). Greater diversity with regard to this parameter was observed only after freezing at –12 °C (Figure S2b). In most cases, the Polish breeding lines and cultivars performed better than the non-Polish European varieties with regard to EL after freezing at –12 °C (Figure S2b).

The most diversifying chlorophyll fluorescence parameters were ET_0_/CS and DI_0_/CS, while F_V_/F_M_ proved to be the least informative as to the level of freezing tolerance among the tested accessions (Fig. 3). Three out of four displayed chlorophyll fluorescence parameters showed better performance of the non-Polish European varieties exposed to freezing (Fig. 3). Only the results of TR_0_/CS were in line with those for EL and FT-R after freezing at − 12 °C (Figure S1b and S2b, Fig. 3b).

### De-acclimation tolerance assessment

Twenty selected lines and cultivars were also subjected to a week in de-acclimating conditions, preceding their freezing tolerance tests. As compared with the control (cold-acclimated) conditions, the de-acclimated accessions showed lower FT-R (Table 3). The biggest difference was observed in EL, which was several times higher in the de-acclimated plants (Table 3).

**Table 3:** Rankings (RNK) of freezing tolerance after de-acclimation measured as plant appearance FT-R (0 – 9 score), % of EL after freezing at -10°C and selected chlorophyll fluorescence parameters measured after freezing of detached leaves. Accession numbers are explained in Table 1. Means are given together with confidence interval limits for *P* = 0.05 according to ANOVA analysis. Lower (-95%) and upper (+ 95%) limit is presented in the case when the highest frost resistance corresponds to a higher or lower value of the parameter, respectively. As a consequence means further down the ranking which are below or above the limit, respectively, did not differ statistically at *P* = 0.05 from the mean which this limit accompanies.

RNK	FT-R [0 – 9]			EL [%]			ET_0_/CS			TR_0_/CS			DI_0_/CS			F_V_/F_M_			RC/CS_M_		
	No	Mean	− 95%	No	Mean	+ 95%	No	Mean	− 95%	No	Mean	− 95%	No	Mean	+ 95%	No	Mean	− 95%	No	Mean	− 95%
1	53	4.10	3.20	41	49.4	67.2	51	106.4	73.1	53	266	217	40	95	131	40	0.72	0.66	6	415	352
2	2	3.59	2.66	42	50.2	67.4	53	94.4	64.0	51	266	218	48	111	148	48	0.70	0.62	51	411	298
3	43	3.41	2.57	38	53.0	72.7	57	87.1	56.1	8	260	228	41	119	161	41	0.69	0.61	31	395	310
4	41	3.37	2.72	40	55.1	71.0	2	85.7	44.0	57	257	212	57	141	196	57	0.66	0.57	41	373	246
5	35	2.93	2.19	14	60.0	83.9	40	80.4	70.7	31	252	223	42	144	199	42	0.65	0.56	8	358	249
6	38	2.92	2.17	48	60.4	84.6	41	79.6	65.5	2	252	203	43	148	214	43	0.643	0.53	38	351	258
7	42	2.85	1.98	8	61.6	80.6	8	78.4	52.9	38	247	219	14	151	206	14	0.64	0.56	40	349	223
8	31	2.79	2.11	35	62.4	75.8	48	77.5	57.2	41	247	234	31	157	189	53	0.63	0.54	48	329	243
9	48	2.76	1.97	53	69.2	90.6	43	74.7	62.8	48	246	226	38	162	211	51	0.63	0.51	43	323	252
10	6	2.60	1.46	31	71.5	87.6	35	69.6	52.9	14	244	222	35	162	225	35	0.62	0.52	2	321	190
11	14	2.58	1.81	43	72.2	91.1	38	68.5	55.2	35	242	215	51	171	234	31	0.62	0.56	53	317	240
12	40	1.71	1.17	47	77.4	90.5	4	66.5	49.2	42	239	216	53	172	242	38	0.62	0.55	14	311	250
13	57	1.51	0.84	51	78.1	96.8	9	64.9	51.1	9	238	219	4	175	240	8	0.61	0.50	4	308	195
14	47	1.50	0.88	57	79.4	93.8	52	63.7	40.3	43	236	211	47	180	247	4	0.58	0.46	57	305	256
15	9	1.47	0.73	7	81.6	97.6	31	59.9	49.3	40	234	214	8	181	256	47	0.57	0.47	42	298	181
16	4	1.36	1.03	52	89.5	106.3	6	59.8	49.6	6	230	198	52	209	302	2	0.56	0.44	47	284	173
17	8	1.32	0.26	9	89.5	101.1	42	58.0	44.7	4	225	194	2	216	294	9	0.56	0.44	9	272	189
18	52	1.10	0.01	6	89.9	99.1	14	57.6	40.6	52	224	184	9	223		52	0.55	0.41	7	268	191
19	51	0.84	0.49	4	95.1	102.0	47	55.6	43.7	47	221	191	6	235		6	0.52	0.41	52	257	193
20	7	0.38	0.06	2	96.6	100.9	7	49.9	37.8	7	204	173	7	243		7	0.49	0.36	35	233	141

**Figure 4:**
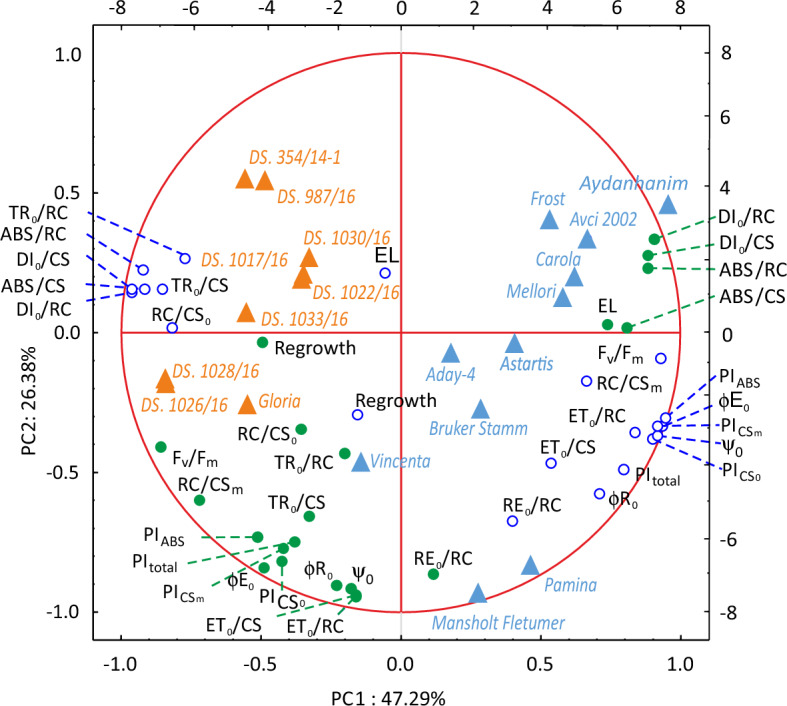
Biplot showing PCA analysis results of variables measured in 20 barley accessions cold-acclimated for 3 weeks at 3 weeks at 4/2 °C, day/night (open, blue dots) and de-acclimated 7 days at 12/5 °C, day/night (closed, green dots) plants as well as the distribution of the accessions (triangles). Polish breeding lines and cultivar are indicated in orange and European cultivars in blue.

The PCA showed that the parameters of chlorophyll fluorescence after de-acclimation were grouped opposite to the same parameters measured in the cold-acclimated plants (Fig. 4), proving that they reflected progress of de-acclimation, and were suitable for selecting barley accessions tolerant to de-acclimation. EL values for the cold-acclimated and de-acclimated plants were also grouped at a distance from each other, but not completely opposite (Fig. 4). FT-R, on the other hand, seemed to identify the plants the same way, regardless of their cold-acclimation status, as the values of this parameter in de-acclimated and non de-acclimated plants were situated near each other on the PCA graph (Fig. 4). This observation was also confirmed by the correlation coefficient for the survival rate of the cold-acclimated and de-acclimated plants, which amounted to 0.616 (data not shown).

All of the Polish new breeding lines, as well as the only Polish cultivar among the tested European varieties, cv. ‘Gloria’, were grouped together with a visible distance from the rest of the studied objects in the PCA graph (Fig. 4). Those lines and cv. ‘Gloria’ showed the best performance after de-acclimation followed by freezing in most of the measured parameters (Table 3). Also the worst overall performing cultivars, namely ‘Aydanhanim’, ‘Astartis’, ‘Avcii 2002’, ‘Carola’, ‘Frost’, and ‘Mellori’ were grouped together in the PCA, suggesting a similar mechanism of response to de-acclimation (Fig. 4, Table 3).

Among three cultivars originating from Turkey, cvs. ‘Avci 2002’ and ‘Aydanhanim’ were grouped together in the PCA, suggesting a similar response to de-acclimation, while cv. ‘Aday-4’ was located in a different quarter of the graph (Fig. 4). These results are in line with FT-R and chlorophyll fluorescence values after de-acclimation of those cultivars, as cvs. ‘Aydanhanim’ and ‘Avci 2002’ were among the worst, while cv. ‘Aday-4’ was among the best objects in terms of FT-R and some fluorescence parameters (Table 3). Cv. ‘Aday-4’ was also the most distinct among all the 20 tested objects. It demonstrated the second best FT-R and the worst EL at the same time (Table 3), and in the PCA graph it was located the nearest to the group of cultivars with the poorest performance after de-acclimation (Fig. 4). Two German cultivars, ‘Pamina’ and ‘Vincenta’, were located quite far from each other in the PCA graph, suggesting their partly different response to de-acclimation. According to the PCA, regarding its reaction to de-acclimation, cv. ‘Vincenta’ seemed to have more in common with the Polish lines and cv. ‘Gloria’ (Fig. 4).

Cvs. ‘Pamina’ and ‘Bruker Stamm II’ were the only cultivars with facultative growth habit tested for their de-acclimation tolerance in this study. They were both located in the same part of the PCA graph, but not close to each other (Fig. 4), suggesting a partially different mechanism of response to de-acclimation. Cv. ‘Pamina’ performed much better than cv. ‘Bruker Stamm II’ in terms of FT-R and some chlorophyll fluorescence parameters, especially ET_0_/CS (Table 3). Most of the lines and cultivars that performed the best in terms of their FT-R after de-acclimation were also among those with the highest ET_0_/CS (Table 3).

### LT50 assessment

**Figure 5:**
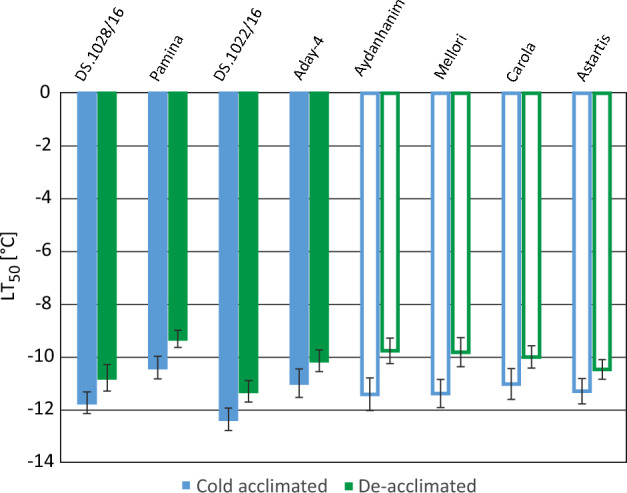
Freezing tolerance expressed as LT_50_ (temperature which killed 50% of the plants) of eight barley accessions (Polish breeding lines and European cultivars) selected for their diverse tolerance to de-acclimation. Tolerant lines were marked with solid and susceptible with open bars. Freezing tolerance was studied after cold acclimation (3 weeks at 4/2 °C, day/night) and de-acclimation (7 days at 12/5 °C, day/night). 50% of plants killed by freezing temperature corresponds to 4.5 score on 0–9 scale of FT-R assessment. Means are given together with 95% confidence limits according to dependent variable prediction in ‘Multiple regression’ module of Statistica 13 (Dell, Round Rock, TX). Means, which are below or above the vertical bar limit did not differ statistically at *P* = 0.05.

LT50 for de-acclimated plants was significantly higher than for cold-acclimated ones (Fig. 5), which confirmed that all studied lines successfully de-acclimated during one week of de-acclimation. The results of LT50 assessment were in line with those of the physiological measurements and FT-R only in the case of two Polish breeding lines (Figs. 4 and 5), confirming their superior freezing and de-acclimation tolerance. On the other hand, the results of LT50 assessment after de-acclimation showed no significant differences between the cultivars selected as susceptible (‘Astartis’, ‘Aydanhanim’, ‘Carola’, and ‘Mellori’), and two of the accessions selected as tolerant (‘Aday-4’ and ‘DS. 1028/16’). The susceptible accessions performed slightly worse than ‘DS. 1022/16’, which showed the lowest LT50 after de-acclimation. Cv. ‘Pamina’, selected as tolerant to de-acclimation in previous analyses, showed the highest LT50 in the conditions of this experiment (Fig. 5).

## Discussion

Although the problem of mid-winter de-acclimation is becoming increasingly crucial for overwintering of herbaceous plants, and future predictions show that its role will only grow in the next decades^1,2,19^, there is still very little known about the mechanisms of response to this stress, both on the phenotypic and genetic level. The existing studies focus mostly on dicotyledonous species^20–23^, especially *Arabidopsis thaliana*^24–28^. What is more, the studies up to now represent a whole spectrum of different de-acclimation times, temperatures, and day lengths, as well as different freezing conditions^20,21,23–25,28–31^. All of the above make mid-winter de-acclimation studies, and comparisons between them, very challenging. Our study used a wide spectrum of physiological indices in order to investigate freezing tolerance in control conditions (cold-acclimated plants), and active de-acclimation tolerance defined here as freezing tolerance of de-acclimated plants. In the freezing tolerance assessment, the results of only one of the fluorescence parameters, namely TR_0_/CS, were in line with the results of EL and FT-R, and indicated the same accessions as the most and least tolerant, while the results for ET_0_/CS and DI_0_/CS were opposite. F_V_/F_M_, which is one of the most widely used chlorophyll fluorescence parameter for assessing freezing tolerance^32–35^, did not differentiate the accessions used in this study, and thus could not serve as a freezing tolerance indicator. Limited usefulness of F_V_/F_M_ as an indicator of freezing damage was reported previously^36^. On the other hand, F_V_/F_M_ was among the parameters that were affected by active de-acclimation in our study, and showed diversity of the investigated barley accessions, which was consistent with the results of FT-R after de-acclimation. The chlorophyll fluorescence parameter that was the most reliable in pinpointing the objects the most and least tolerant to de-acclimation, was ET_0_/CS. These results confirm that ET_0_/CS is one of the most useful chlorophyll fluorescence parameters, usually the best in terms of correlating with survival after freezing in laboratory and field conditions^37,38^.

De-acclimation affected mostly EL from the leaf tissues, when compared with the chlorophyll fluorescence parameters and FT-R. Dramatically increased EL in de-acclimated plants, as compared with the cold-acclimated ones, indicated that the changes in response to de-acclimation appear sooner in the leaves (but not directly in the photosynthetic apparatus) than in the other parts of the plant. This type of reaction was especially visible in one of the most de-acclimation tolerant cv. ‘Aday-4’, which displayed the highest EL among all the studied accessions. Apart from EL, also the chlorophyll fluorescence parameters related to the membrane integrity, namely DI_0_/CS and RC/CS_M_, showed the greatest changes after de-acclimation in most of the studied accessions. A possible reason for high survival rate in de-acclimated plants with apparent severe membrane damage in the frozen leaves, could be their ability to regenerate from the tillering nodes, as suggested previously^39,40^.

However, there are some accessions tolerant to de-acclimation, e.g. ‘DS. 1022/16’ and ‘DS. 2026/16’, in which de-acclimation seems to affect EL and FT-R to a similar degree. This may mean that the mechanisms of de-acclimation tolerance are different in different barley accessions. A similar conclusion was drawn in our previous study^9^, where it was proposed that the response to de-acclimation in the susceptible accessions is probably similar, while the mechanisms of tolerance to de-acclimation are difficult to identify due to their diverse nature. Common mechanisms of response to de-acclimation in the susceptible barley accessions seem to be also confirmed by the PCA, in which the most susceptible accessions were grouped together despite their different origin.

The results of LT50 assessment for cold-acclimated and de-acclimated plants correlated with those for freezing and de-acclimation tolerance only to some extent, namely in the case of the Polish breeding lines selected as tolerant. A direct comparison of LT50 and both types of freezing tolerance assessment used in this study is of course impossible. That is mainly due to different methods used. The freezing tolerance assessments, both for the cold-acclimated and de-acclimated plants, included EL, chlorophyll a fluorescence measurements, and FT-R tests, and the overall freezing and de-acclimation tolerance assessment took all those results into account. On the other hand, in LT50 assessment, only FT-R test was performed. That is also an argument in favor of the hypothesis that the de-acclimation-related changes appear first in the leaves of a frozen plant. The observed differences might also be caused by a slightly different freezing protocol used in those experiments. In the case of LT50 assessment, the freezing time was shorter than in the preceding experiments, as we needed to test many more freezing temperatures in a single experiment. The differences in freezing tolerance of the cold-acclimated and de-acclimated plants, observed between the LT50 test and the other tests, confirmed the role of freezing exposure time in inducing plant damage^41–43^.

The Polish breeding lines and cultivars performed better regarding FT-R and EL both after cold acclimation and de-acclimation. However, the other European cultivars displayed better photosynthetic performance after freezing, as measured by chlorophyll fluorescence parameters in cold-acclimated state. After de-acclimation, the Polish accessions showed higher tolerance of the photosynthetic apparatus to direct influence of freezing, as evidenced by the values of chlorophyll fluorescence parameters. These results show extreme vulnerability of the photosynthetic apparatus to direct freezing in the de-acclimated state, which is in line with the previous suggestions on the role of the photosynthetic apparatus in de-acclimation signal perception^21^, as well as the role of the antioxidant system in the response to active de-acclimation^9^. The differences in overall performance and the fact that the Polish breeding lines and cv. ‘Gloria’ form a distinct group in the PCA of the performance after de-acclimation, might indicate that the origin of barley accessions determines their reaction to de-acclimation. Different origin, as well as breeding and cultivation area, might have created an unintended selection pressure, of which the breeders were unaware. Thus, the genetic background of the response to mid-winter active de-acclimation might be different in the cultivars of various descent. These alleged differences might be responsible for the tolerance mechanisms observed in this and our previous study^9^.

In conclusion, the differences in freezing tolerance and tolerance to de-acclimation of the tested accessions support the hypothesis that these two traits are determined separately, and even highly freezing tolerant plants can be susceptible to de-acclimation. Our results highlight the role of the photosynthetic apparatus in the de-acclimation process, proving that the chlorophyll fluorescence parameters, especially ET_0_/CS, can be useful indicators of tolerance to de-acclimation, and thus help in the selection process. This study also confirmed our previous findings, that although the mechanisms of response to de-acclimation seem common for the susceptible barley accessions, the mechanisms of tolerance are different and may be related to the plant origin.

### Supplementary Information

Supplementary Information 1.

Supplementary Information 2.

## Data Availability

The datasets generated during and/or analyzed during the current study are partly available as supplementary materials (Figures S1 and S2), and partly from the corresponding author on reasonable request.
