# Supplementary material for: Freezing tolerance and tolerance to de-acclimation of European accessions of winter and facultative barley
Source: Sci Rep. 2023 Nov 15;13:19931. doi: 10.1038/s41598-023-47318-y (PMC10651919; doi:10.1038/s41598-023-47318-y)
Supplement: Supplementary file 1 — Supplementary Information 1. [file 41598_2023_47318_MOESM1_ESM.docx]

a)

b)

Fig. S1. Plant regrowth after freezing (FT-R, scale 0-9) in a) -8 °C and b) -12°C. Means ± confidence intervals for P=0.05. Homogeneity groups (HSD test, P=0.05) for panel b (freezing at -12°C are shown in Table S1. In the case of panel a (freezing at -8°C) no statistically significant differences between accessions were detected by HSD Tukey’s test for P=0.05.

a)

b)

Fig. S2. Electrolyte leakage (EL) [%] measured on leaves of Polish breeding lines and European cultivars after freezing in a) -8°C and b) -12°C. Means ± confidence intervals for P=0.05. Homogeneity groups (HSD test) are shown in Table S1.
